# Supplementary material for: Assessment of Risk Hospitalization due to Acute Respiratory Incidents Related to Ozone Exposure in Silesian Voivodeship (Poland)
Source: Int J Environ Res Public Health. 2020 May 20;17(10):3591. doi: 10.3390/ijerph17103591 (PMC7277508; doi:10.3390/ijerph17103591)
Supplement: Supplementary file 1 [file ijerph-17-03591-s001.pdf]

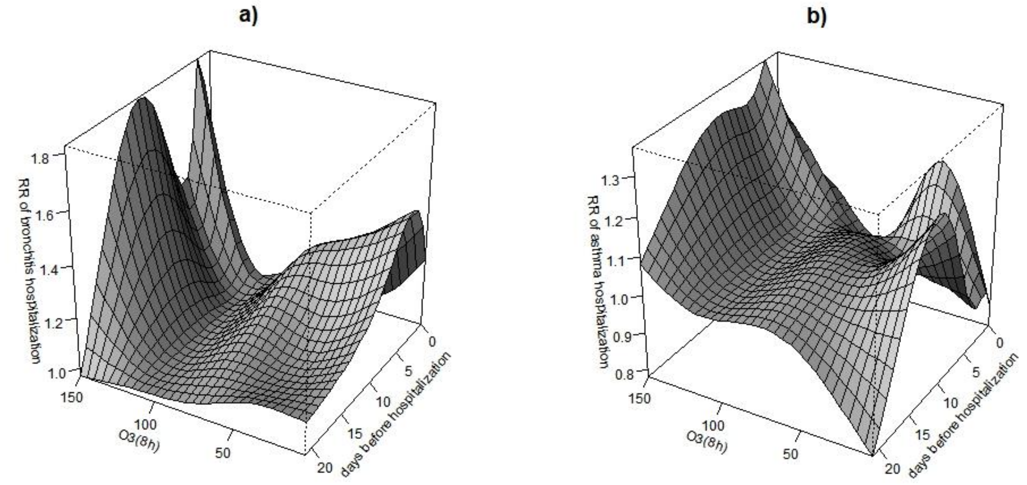

Figure 1. Relative risk RR_l of daily hospitalizations due to: **a**) Bronchitis (J20–J21), **b**) asthma (J45–J46), related to eight-hour O_3 ozone concentration.

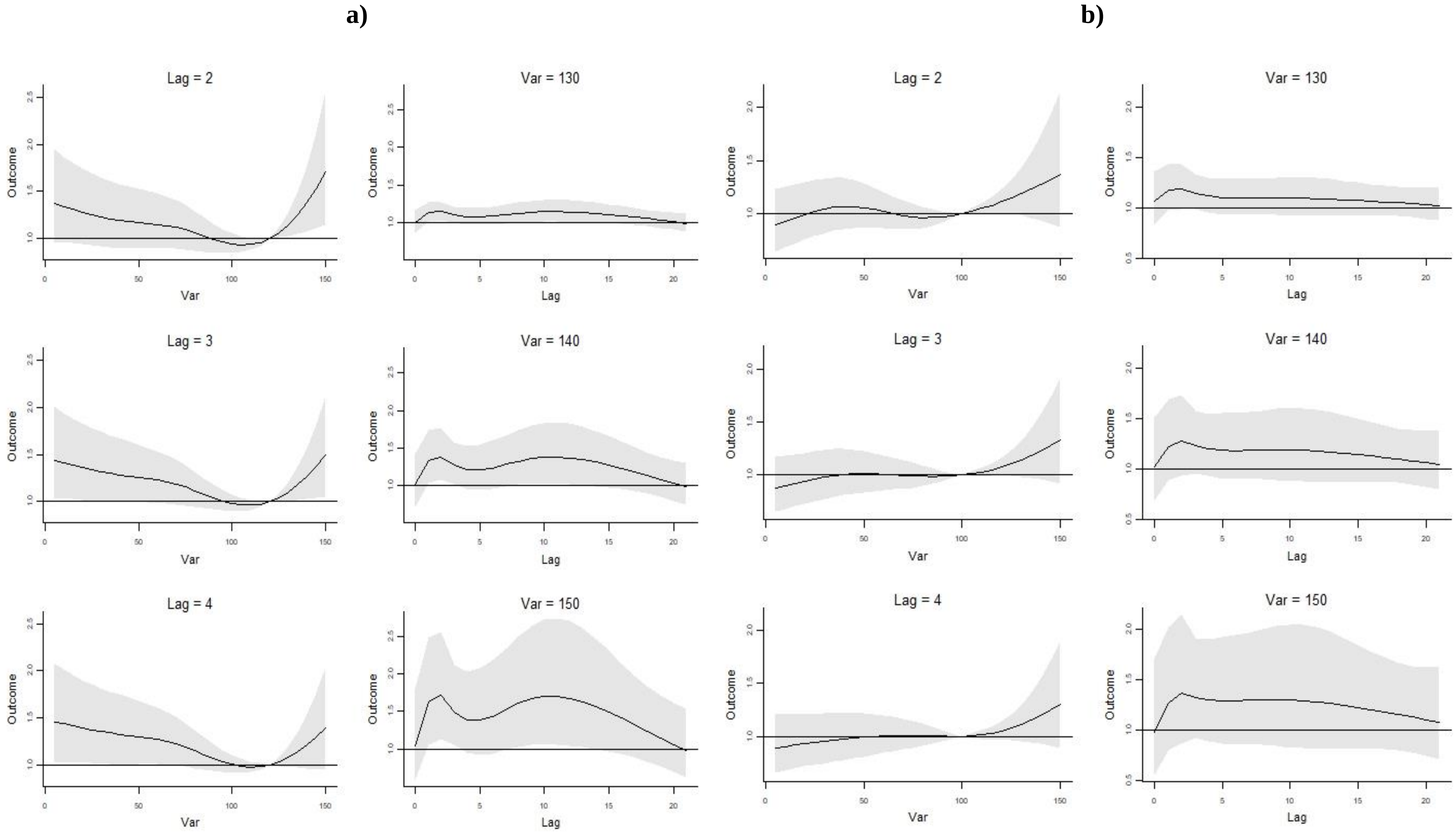

Figure 2. Relative risk RR_l of daily hospitalizations due to: **a**) Bronchitis (J20–J21), **b**) asthma (J45–J46), in the first days after exposure and associated with an 8 h ozone concentration increase by next unit (10 μg/m³) compared to a normative value of 120 μg/m³.
